# Qualified foreign institutional investors and corporate innovation: From the perspective of corporate governance

**DOI:** 10.3389/fpsyg.2022.1005409

**Published:** 2022-11-18

**Authors:** Xiao Wang, Wanting Wang, Xiang yan Shi

**Affiliations:** ^1^The School of Accounting, Southwestern University of Finance and Economics, Chengdu, Sichuan, China; ^2^The School of Accountancy, Shandong University of Finance and Economics, Jinan, Shandong, China

**Keywords:** qualified foreign institutional investors, corporate innovation, “tunnel”, analysts following, corporate efficiency

## Abstract

Whether qualified foreign institutional investors can effectively play a governance role in the capital market and guide the transformation of corporate innovation from “high-volume and low-quality” to “high-volume and high-quality” is an important issue in the process of foreign capital introduction at the present stage. From the perspective of how QFII shareholding affects the innovation model of firms, this study analyzes the data of China’s A-share listed companies from 2007 to 2018 and finds that the shareholding of qualified foreign institutional investors has significantly improved the innovation level of the invested firms, which is reflected in the increase of innovation output and the improvement of innovation quality. The mediating effect shows that QFII shareholding can improve the innovation level of corporates by slowing down insider tunneling of holding companies and increasing the number of analysts to follow, which indicates that QFII is conducive to improving the governance structure of listed companies and improving their qualities. Further research finds that QFII shareholding has a positive impact on corporate efficiency by improving the level of corporate innovation. The above conclusions provide experience and reference for China to further introduce foreign capital.

## Introduction

**Figure 1:**
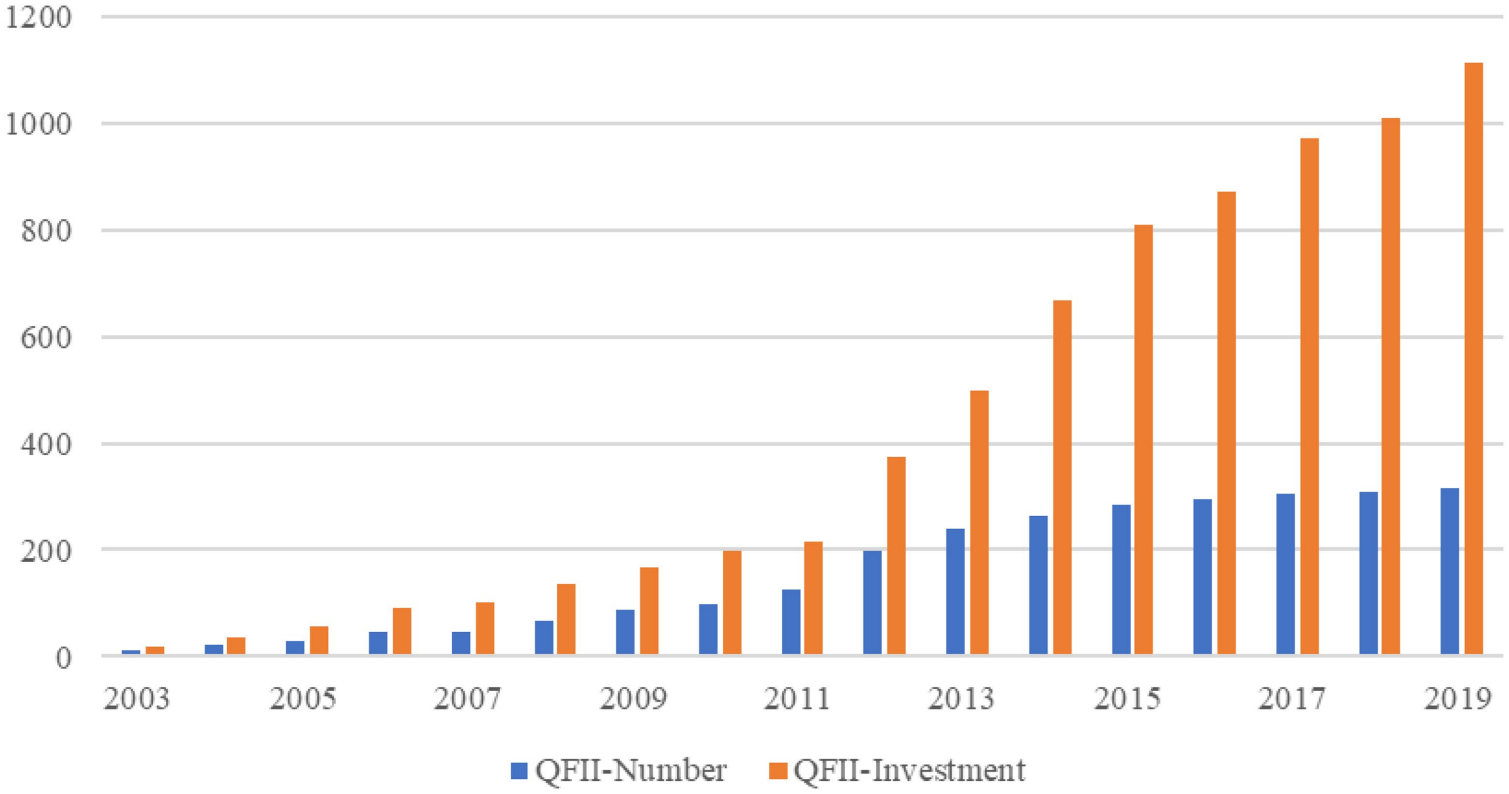
Statistics on the number and investment volume of QFII from 2003 to 2019. Remarks: Quantity unit: individual investment unit: billion US dollars.

The New Growth Theory believes that innovation is the main driving force for long-term economic development (Romer, 1990). As China’s economy enters the “new normal” and a critical period of transformation and development, innovation has become an important way to enhance the value of firms and promote the growth of the national economy. In 2017 the 19th National Congress of the Communist Party of China clearly proposed to accelerate the construction of an innovative country, put innovation at the core of leading development, deepen the reform of the scientific and technological system, and establish a market-oriented technological innovation system with the firms as the main body, and deep integration of production, education, and research. Then, at the Fifth Plenary Session of the 19th Central Committee in October 2020, the requirements for high-level opening up have been put forward, illustrating the importance of achieving a new situation of win–win cooperation. To better integrate with the world capital market, continuously introduced Qualified Foreign Institutional Investors (QFII) since 2003 to actively build a rational, orderly and inclusive capital market. As of the end of 2019, 316 overseas institutions from 31 countries and regions have obtained QFII qualifications, with a total approved quota of US$111.522 billion. The specific amount and quantity are shown in Figure 1. As a transitional arrangement for introducing foreign capital and opening up the capital market, the fundamental purpose of the QFII system is to attract foreign capital into China’s capital market and improve the quality and efficiency of China’s capital market. Existing researches have studied the effect of the QFII system on China’s capital market efficiency and corporate behavior from the aspects of capital market (Liu et al., 2020), corporate finance (Liu and Li, 2022), and corporate governance (Li et al., 2018a,b, 2021). However, little literature has explored the effect of QFII on corporate innovation and the mechanism of effect, but the rational and efficient introduction of foreign capital plays an important role in China’s economic growth, technological progress, and export development (Anwar and Sun, 2014; Chen and Luo, 2014). On this basis, this paper focuses on the effect of QFII on micro-corporate innovation and its potential effect paths.

The impact of QFII on a firm’s behavior is gradually gaining attention, but there is relatively little literature on corporate innovation and the paths of influence. Zhang and Ni (2017) explored the impact of QFII on innovation in terms of how QFII enhances the information content of stock prices and reduces executive compensation-performance sensitivity, where no significant role of QFII in the level of governance was found. In contrast to their paper, the empirical research of this paper finds that the mechanism of QFII’s promotion of corporate innovation is to alleviate the “tunnel” behavior in the internal governance of the firm and improve the degree of analysts following in the external governance. This suggests that QFII has a significant governance effect on Listed Companies in China and can enrich the current mechanism of QFII’s impact on corporate innovation. In addition, this paper attempts to study whether the introduction of QFII can solve the dilemma of “high quantity, low quality” of Chinese innovation from the dual perspective of innovation quantity and quality while incorporating the changes in the internal and external governance environment of firms into the analysis framework, and eventually provide theoretical and empirical support for the concept of innovation and open development proposed at the Fifth Plenary Session of the 19th Central Committee.

Specifically, from the perspective of how QFII affects corporate innovation, this paper uses the data of China’s A-share listed companies from 2007 to 2018 and finds that: (1) QFII shareholding has significantly improved the innovation level of the invested firms, which is manifested in the increase in innovation output and the improvement of innovation quality. (2) The intermediary mechanism inspection finds that QFII shareholding can improve the level of corporate innovation by slowing down insiders’ “tunnel” of the holding company and increasing the number of analysts following, which indicates that QFII can improve the governance structure of the listed companies and improve their quality. (3) QFII shareholding has significantly improved corporate efficiency by enhancing the level of corporate innovation, indicating that QFII can play the role of “value creators” that improve corporate efficiency. The above research results show that China’s QFII system has achieved phased results. The introduction of high-quality investors into the capital market will not only help improve the internal and external governance structure of firms but also improve the level of corporate innovation and promote the high-quality development of China’s real economy.

Compared with previous studies, this paper comprehensively discusses the impact of QFII as an important category of institutional investors on corporate innovation and its effect path in China. The main contributions of this paper are as follows: First, QFII’s research on firm financing constraints and capital market stability has been widely concerned, while QFII’s governance function as a strategic investor has not attracted much attention. From the perspective of improving the internal and external governance environment of firms, this paper studies the impact of QFII on the innovation level of firms, and expands and enriches the research on the economic consequences of QFII. Second, it enriches the research on the contributing factors of corporate innovation. This paper studies the degree of improvement of innovation level from the perspectives of innovation quantity and quality, and proposes new factors affecting corporate innovation. Third, this paper explores the potential paths of QFII shareholding to promote corporate innovation, and finds that QFII shareholding can improve corporate innovation output and quality by strengthening internal supervision and attracting more analysts following. It is of great practical significance to further guide the inflow of high-quality capital and promote the innovation and development of firms under the concept of national open development.

## Literature review and research hypotheses

### Literature review

#### Literature on qualified foreign institutional investors

Qualified Foreign Institutional Investors mostly refers to internationally renowned institutional investment companies, and financial institutions, such as Swiss Bank, Goldman Sachs, JPMorgan Chase Bank, and Goldman Sachs International Asset Management Company, etc., which have rich investment experience, mature fund operation modes, and rational investors who attach great importance to performance, dividend, s and firm growth. Relevant studies have shown that as professional and unique foreign institutional investors, QFII play an important role in governance and supervision in addition to reducing a company’s cost of capital (Bekaert and Harvey, 2000), alleviating its financing constraints, and enhancing its long-term capabilities of debt financing (Deng and Sun, 2014). He et al. (2013) believed that foreign institutional investors would form effective external supervision on the information content of listed companies’ stock prices. Moreover, compared with domestic institutional investors, QFII not only has rich experience in international investment, and professional value investment ideas, but also have independent investment positions (Chen et al., 2007; Kong et al., 2020), because QFII has fewer business contacts, thus they can be free from managers’ constraints and play a more effective role of governance and supervision (Gillan and Starks, 2003). Specifically, Admati and Pfleiderer (2009), Aggarwal et al. (2011) and Liu and Zhao (2021) find that foreign institutional investors can improve corporate governance and influence the decision of the board of directors through voting rights and threats to sell stocks; He et al. (2013) believed that QFII have excellent capital operation capabilities, and can efficiently integrate and analyze information, thus help to alleviate the information asymmetry between firms and the outside world and help firms make more effective investment decisions. Kong et al. (2020) believed that foreign-funded institutions should increase the proportion of R&D expenditure in revenue, limit the scale of related party transactions to improve corporate governance, and widen the pay gap within enterprises to stimulate human capital efficiency. Chen et al. (2014) find that foreign strategic investors can help improve the executive compensation mechanism and encourage managers to improve corporate governance by increasing the performance sensitivity of managers’ compensation. Wang and Cheng (2022) believed that the impact of executive stock ownership on corporate innovation presented an inverted “U” shape; Institutional investors will negatively regulate the impact of executive shareholding on corporate innovation. Li et al. (2018a,b) find that QFII provide listed companies with a special and professional supervision force from abroad, which can enhance the quality of companies’ information disclosure.

However, supervision takes time and cost, and based on cost–benefit considerations, QFII may also only play the role of short-term profit seekers (Callen and Fang, 2013). Because foreign institutional investors have a low degree of localization, the cost of participating in corporate governance is relatively high (Fu, 2008; Areneke et al., 2022). Moreover, they may have little understanding of the national conditions of the investee countries, which makes it so difficult for them to actively participate in the corporate governance of the investment firms that they can only choose passive value investment, and because Chinese firms have strong political power and networks, qualified foreign institutional investors (QFII) are reluctant to strengthen innovation activities (Ain et al., 2021). In addition, the shareholding ratio of QFII is usually low, which leads to their lack of motivation to participate in corporate governance and improve company performance, so they only play the role of “value investors” rather than “value creators” that improve the company performance (Li and Han, 2014). Tan (2009) questionnaire survey on Chinese QFII finds that QFII have neither appointed independent directors nor delegated representatives in investee companies, so it is difficult for QFII to play a governance role. Furthermore, compared with domestic institutional investors, the average investment amount of a single QFII company is much lower than that of domestic institutional investors. From the perspective of cost and benefit, this will further depress the enthusiasm of QFII to participate in corporate governance and improve company performance. Feng and Wen (2013) find that QFII have almost no inhibitory effect on related party transactions. The above factors also lead to QFII’s strong value selection ability, but their value creation ability is not reflected (Tang and Song, 2010). Chi et al. (2019) believed that the shareholding of qualified foreign institutional investors (QFII) had little or no significant impact on innovation. To sum up, there is still no conclusion about the influence of QFII on the overall governance effect.

#### Related literature on corporate innovation

Innovation activities are naturally characterized by high risk and long cycles, which may increase the degree of information asymmetry and agency problems of firms, thereby reducing shareholders’ willingness to invest. Specifically, first, the high risk and uncertainty of R&D innovation activities will increase the degree of information asymmetry of firms. The first cause is that when investors do not understand the innovative technology that the company wants to invest in, they will be reluctant to invest because it is difficult to evaluate its value (Graham et al., 2005; Feng and Wen, 2008; Shaikh and Randhawa, 2022). Besides, firms are reluctant to fully disclose information on innovative projects to external investors due to concerns about the leakage of innovative technical information and the imitation of competitors. On this basis, the problem of information asymmetry between the two parties has led to a reduction in shareholders’ willingness to invest (Bhattacharya and Ritter, 1983; Baruffaldi et al., 2022). Secondly, innovation activities are also characterized by long cycles, in which innovative projects take years from inception to progress to generating value, during which management may get questioned for the low effectiveness of short-term innovation (Lu et al., 2020). Manso (2011) and Ederer and Manso (2013) argued that the process of innovation activities can distort the consistency of objective functions between management teams and shareholders and that managerial decisions may work against shareholders during the R&D process.

From the perspective of improving the factors affecting the internal and external governance environment of corporate innovation, the existing literature on the internal governance environment mainly examines the impact of equity structure, equity pledge, director network, compensation gap, equity incentive, executive background, *d* and institutional investors (Wen and Feng, 2012; Li and Yu, 2015; Kong et al., 2017; Liu and Wang, 2018; Wang and Zhang, 2018; Yu et al., 2018; Li et al., 2018a,b; Hao et al., 2020; Peng et al., 2020). For example, Li et al. (2018a,b) find that, from the perspective of controlling shareholder pledges, controlling shareholder equity pledges will inhibit corporate innovation investment, and this negative impact will only play a role when the equity pledge rate is high and the distance from the liquidation line is closer. Aghion et al. (2013) find from a supervisory perspective that the higher the shareholding ratio of institutional investors, the more conducive to improving the level of innovation of firms. Wen and Feng (2012) and Huang and Zhu (2015) find that the negative effect of securities investment funds on corporate innovation is more significant in state-owned firms, while institutional investors in private firms promote the development of their innovation activities. In terms of the external governance environment, the existing literature mainly examines the influence on corporate innovation from government systems, industrial policies, analysts following, etc. (Gu and Shen, 2012; Chu et al., 2016; Yu et al., 2016; Chen et al., 2017; Guo, 2018; Wang et al., 2018; Dai et al., 2019). Chen et al. (2017) discuss the positive impact of the number of analysts following corporate innovation from the perspective of analysts’ information transfer and professional ability.

### Research hypothesis

In the context of the current economic transformation in China, “innovation” has become a hot topic in the research of macro economy and micro enterprises. According to previous literature, the main reasons that affect the innovation output are: based on information asymmetry or agency problems, corporate management, or major shareholders having short-sighted or usurped interests. In addition, innovation itself has the characteristics of a long cycle, high risk, and high uncertainty, and the attribution of its success or failure becomes harder (Holmstrom, 1989). In other words, the long process of R&D, is usually accompanied by greater difficulty and uncertainty, which makes it difficult to accurately evaluate the value of enterprise innovation activities. It also urges different investors to have a different understanding of the risk–return generated by the innovation value of enterprises. The entry of QFII can not only directly provide financial support for enterprises, but also convey the signal of high enterprise value and good development potential to the market. According to the effect of QFII supervision and governance and signaling effect, QFII usually has the governance effect of reducing information asymmetry and agency problems, as well as the signaling effect of attracting the attention of external analysts and stakeholders, which can positively promote enterprise innovation.

Specifically, on the one hand, QFII investment decision-making usually has a complete and detailed process, including in-depth research before investment, ongoing follow-up, supervision, and investment summary after the event. At the same time, the concept of QFII long-term value investment has strongly driven the professional investment management team to function as supervision and governance. By reducing information asymmetry and agency problems in the process of capital use, the efficiency of capital use can be improved and more innovation output can be generated. Institutional investors can exert pressure on companies to promote them to adopt better accounting policies, and also reduce the level of information asymmetry between companies and the outside world by publishing the earnings management of their shareholding companies. Li et al. (2018a,b) found that QFII shareholding can improve the quality of information disclosure of Chinese listed companies. In combination with the inspection of the internal and external environment of enterprises, QFII has provided a special and professional supervision force for companies from abroad. However, enterprise innovation is a specialized activity with a high degree of information asymmetry. QFII is capable of improving the quality of information and reducing the level of information asymmetry, which can avoid the underestimation of the company’s innovation level, reduce the probability of misjudgment of innovation attribution, and thus enhance the company’s enthusiasm for innovation.

On the other hand, QFII investment has the function of signal transmission, releasing the signal of enterprise innovation potential, attracting interested parties (such as analysts, government, media, etc.) to increase their attention and analysis of enterprise behavior from different levels, helping investors to identify the investment value of enterprise innovation activities, and generating some “spillover effects.” Similarly, this “spillover effect” will, to a certain extent, solve the problems of information asymmetry and financing constraints in the process of enterprise innovation activities, thereby enhancing the willingness and ability of enterprises to innovate and improve their innovation output. As a qualified foreign institutional investor with an information governance function (Li et al., 2018a,b), its investment behavior will have a direct impact on information analysts. Because QFII has strict procedures before, during, and after the investment process, this process will produce a certain spillover effect, which can convey to the market what kind of enterprise is a better enterprise, thus attracting more interests related attention and tracking. Further combined with the spillover effect of signal transmission, the entry of QFII can not only directly reduce the financing constraints of enterprises but also attract more investors and ease the financing constraints of enterprises by reducing the level of information asymmetry in the process of enterprise innovation When the level of enterprise information asymmetry is relatively high), they usually face higher financing costs (Myers and Majluf, 1984). Qualified foreign institutional investors can play the role of information transmission at a certain level to ease the level of information asymmetry between enterprises and investors. By improving the quality of enterprise information, they can reduce the company’s financing costs and alleviate the shortage of innovative funds, and then achieve the effect of improving the level of enterprise innovation.

Based on the above analysis, we propose the following assumptions:

*H1*: Qualified foreign institutional investors can effectively promote corporate innovation, which is embodied in increasing the innovation output of firms and improving the innovation quality of firms.

Compared with enterprises in mature capital markets, Chinese listed companies have a common phenomenon of “sole majority shareholder” due to their special historical and institutional background (Hou et al., 2017); However, the external investors of listed companies in China are mainly retail investors, and their power and ability to supervise major shareholders of enterprises are very limited, which makes the second type of agency problem in corporate governance more obvious, specifically, large shareholders encroach on the company’s assets for their interests, thus damaging the interests of small and medium-sized shareholders, namely “tunneling” (Johnson et al., 2000; La Porta et al., 2000). The “tunneling” behavior of enterprises is usually a mismatch of enterprise resources, which will produce a crowding out effect on innovation funds, reduce the resource allocation of enterprise innovation and affect the improvement of enterprise innovation level. Specifically, first, the failure of innovation activities may be used as an important means to cover up the “tunneling” behavior of large shareholders, and it is not easy to be found by small and medium-sized shareholders (Chen et al., 2017). Second, the innovation output may also be plundered by major shareholders, thereby reducing the enthusiasm for internal innovation (Gavious et al., 2015).

According to the theory of corporate governance, as a special shareholder group, QFII has rich investment experience and a professional investment team, and has the advantage of economies of scale in terms of management cost and information acquisition (Chemmanur et al., 2009; Lel, 2019), to play the role of supervision and governance to promote innovation. QFII is usually able to use its professional analysis, research, and face-to-face communication with major shareholders to monitor the “tunneling” behavior of enterprises, reduce the interest encroachment of major shareholders and enhance innovation investment with long-term value. QFII can effectively analyze, discover and identify the “tunneling” behavior in the internal economic activities of enterprises. At the same time, foreign institutional investors can exert pressure by “voting with their feet.” When enterprises are found to have “tunneling” behavior, foreign institutional investors can exert pressure on the company by selling shares to urge the company to adjust its improper behavior. Meantime, it reminds shareholders and the board of directors to pay attention, and then exerts pressure on operators to promote their long-term development and avoid short-term opportunistic behavior, thus reducing the occurrence of “tunneling” behavior of major shareholders. QFII, which is heavily held, can supervise the invested enterprises more effectively and mitigate the negative impact of management’s short-sighted behavior on enterprise innovation. Moreover, combined with the concept of long-term value investment of qualified foreign investors, it can reduce the “tunneling” behavior of enterprises. At the same time, it is more conducive to guiding capital inflow into innovation activities with long-term value orientation and increasing the innovation output of enterprises.

Based on the above analysis, we propose the following assumptions:

*H2*: Qualified foreign institutional investors can increase the number of enterprises' innovations and improve the innovation quality of enterprises by improving the "tunneling" behavior of major shareholders in China.

Analysts are both information users and information transmitters, which can play a role in reducing the degree of information asymmetry between market participants, increasing the transparency of information, and supervising the opportunistic behavior of managers in the capital market. Enterprise innovation is characterized by high risk. Most of the relevant innovation information involves trade secrets, which makes it impossible for external investors to fully grasp the information about relevant innovation behaviors, leading to moral hazard and adverse selection problems for enterprises. Especially in reality the governance structure of Chinese listed companies is not perfect and the information transparency is generally low, it provides a broader space for analysts to play the role of information interpretation and supervision.

As a mature and stable qualified foreign institutional investor, the enterprises invested by QFII are likely to receive more attention from the market, which will make external interested parties pay more attention to the behavior of such enterprises and analyze them. In addition, with the strengthening of the supervision role of external third parties such as analysts in China’s capital market, they can better independently understand and discover the intrinsic value of enterprise innovation and reduce the degree of information asymmetry. Specifically, analysts, as an important force for the orderly development of the capital market, will have an important intermediary effect in the process of introducing foreign capital. First of all, from the impact of QFII on analyst tracking, QFII investment itself has a signal transmission mechanism. The “spillover effect” triggered by the preciseness of its investment behavior can help analysts to capture more high-quality enterprises in the market and continue to follow and track them. Secondly, from the relationship between analysts and innovation itself, analyst tracking can improve the innovation level of enterprises. Because analysts usually track specific enterprises and related industries for a long time, some analysts also have relevant professional backgrounds and can better understand and discover the intrinsic value of enterprise innovation. At the same time, as an independent third party, analysts can objectively and impartially convey innovation-related information to the market, reduce agency problems in the innovation process, improve the understanding of external investors on enterprise innovation and increase the innovation level of enterprises. When QFII investment attracts more analysts’ attention, the information asymmetry and governance problems generated in the process of enterprise innovation will be better improved and enhanced, which can significantly improve the innovation level of enterprises.

Based on the above analysis, we propose the following assumptions:

*H3*: Qualified foreign institutional investors can increase the number of enterprises' innovations and improve their innovation quality by attracting more analysts to track.

In summary, we believe that QFII with value investment can effectively curb the “tunnel” of major shareholders and play a corporate governance role. It can also attract more analysts to follow, and under the role of signal transmission, raise the common attention to the innovation activities of firms. Based on the improvement of the internal and external environment of the firm, the quantity and quality of corporate innovation will be significantly improved.

## Research design and descriptive statistics

### Data sources and sample selection

The data of qualified foreign institutional investors (QFIIdum and QFIIshare) in this article mainly comes from the “Institutional Investor” sub-database in Guotai Junan (CSMAR), the corporate innovation data (Ln_Invtotal and Ln_Invia) comes from the “Innovation Patent Research” sub-database in the China Research Data Service Platform (CNRD), and other variable data are from the Guotai Junan (CSMAR) database. The research samples are all A-share listed companies listed on the Shanghai Stock Exchange and Shenzhen Stock Exchange. Considering the impact of China’s newly formulated accounting standards in 2006 on the measurement of firm accounting indicators and foreign institutional investors, the sample interval selected in this paper is 2007–2018. Further, we screened the samples according to the following criteria: (1) excluding financial industry samples; (2) excluding samples with special treatment (ST); (3) excluding samples with missing data, and finally obtained 21,301 firm-year observations. At the same time, to reduce the influence of variable outliers on the research conclusions, we abbreviated the relevant continuous variables in the model at the 1 and 99% levels.

### Variable definition

#### Explained variable: Corporate innovation (innovation: Ln_Invtotal, Ln_Invia)

Innovation_i,t_ is the dependent variable of the model, representing the corporate innovation of firm *i* in year t. This paper measures corporate innovation from the perspectives of total innovation quantity and innovation quality. According to the national patent law, China’s patents include three categories: invention patents, utility model patents, and design patents, of which invention patents are the most original. Therefore, drawing on the research of Chen et al. (2016), this paper measures corporate innovation from the perspectives of its total quantity and quality. The number of invention patent applications (Inviait) is selected as the agent variable of innovation quality, and the total number of patent applications (Invtotalit) is used as the agent variable of the number of innovations. To solve the sample skewness problem, this paper performs natural logarithmic processing after adding 1 to both Indextotalit and Inviait indicators, that is, using Ln_Invtotalit and Ln_Inviait to measure the innovation level and quality of firms.

#### Explanatory variables: Qualified foreign institutional investors(QFII: QFIIdum, QFIIshare)

QFIIdum is a model explanatory variable that reflects QFII’s impact on innovation about whether a firm has QFII or not. If the firm has qualified foreign institutional investors in the year, the value of this variable is 1, otherwise, it is 0. Furthermore, considering that the level of ownership by qualified foreign institutional investors may vary in the degree of impact on corporate innovation, we used the qualified foreign institutional investor shareholding ratio (QFIIshare) to measure its impact on innovation, drawing on the research of Zhang and Ni (2017).

#### Control variable

Referring to the research of Chen et al. (2016), and Zhang and Ni (2017), this paper mainly selects the following control variables in terms of firm characteristics and corporate governance: Size (firm size) refers to the natural logarithm of total assets at the end of the period, Lev refers to the asset-liability ratio, Roa refers to the return on assets, Growth refers to the operating income growth rate. Board refers to the size of the board of directors, Indep refers to the proportion of independent directors, Age refers to the age of the firm, and CFO refers to the cash flow generated from total operating activities. Concerning the research control variables of Jiang et al. (2020), the shareholding ratio of the largest shareholder (Top1) and the nature of the firm (Soe) are also added. Referring to existing literature (Chen et al., 2017), the control variable also selects the number of analysts to track (Follow). In addition, this paper also regulates fixed year effect (Year) and industry fixed effects (Industry). See Table 1 for specific variable definitions.

**Table 1:** Definition of main variables.

Variable name	Variable definition
QFIIdum	Qualified foreign institutional investors, 1 = Qualified foreign institutional investors exist, 0 = No qualified foreign institutional investors
QFIIshare	The shareholding ratio of qualified foreign institutional investors, the shareholding ratio of QFII to the total shares of the listed company
Ln_Invtotal	Innovation output, Ln_Invtotal = Ln (1 + total number of patent applications)
Ln_Invia	Innovation quality, Liana = Ln (1 + total number of invention patent applications)
RPTratio	Firm hollowing-out related party transactions, excluding the sum of annual related party transactions in the five types of transactions, including cooperation projects, licensing agreements, research and development results, remuneration of key management personnel, and other matters
Follow	Number of analyst tracks, Follow = natural logarithm of the number of analyst tracks +1
Size	Firm size, the natural logarithm of total assets at the end of the period
Lev	Gearing ratio, total liabilities/total assets
Roa	Return on total assets, net profit/total assets at the end of the period
Growth	Operating income growth rate, (current operating income—last period operating income)/current operating income
CFO	Company cash flow, net cash flow from operating activities/total assets at the end of the period
Top1	Shareholding concentration is the shareholding ratio of the largest shareholder
Dual	The concentration of management power, whether the chairman and the general manager are combined into one
Board	The number of board members plus 1 takes the natural logarithm
Indep	The proportion of independent directors, number of independent directors/total number of board members
Soe	The nature of property rights, 1 = state-owned firm, 0 = non-state-owned firm
Age	The age of the listed company, the number of years of listing of the firm plus 1 to take the natural logarithm

### Model design

Principal regression model: to test the impact of qualified foreign institutional investors (QFII) on corporate innovation, We constructed The following model, namely model (1), To test The main hypothesis of this paper:


(1) $$\begin{array}{l} {\mathrm{Innovation}}_{i,t}=\beta_{0}+\beta_{1}\times {\mathrm{QFIIdum}}_{i,t}/{\mathrm{QFIIshare}}_{i,t}\\ \;+{\mathrm{Controls}}_{i,t}+\mathrm{Year}+\mathrm{Industry}+\varepsilon \text{.} \end{array}$$

Based on the principal regression model, we further examined the intermediary effect between qualified foreign institutional investors and corporate innovation, and according to the theoretical deduction of the above, we mainly examined the reduction of the internal “tunnel” behavior of the firm and the increase in the number of external analysts following. Based on the model (1), we further constructed models (2) and (3) to test the mediation mechanism.


(2) $$\begin{array}{l} {\mathrm{RPTratio}}_{i,t}/{\mathrm{Follow}}_{i,t}=\beta_{0}+\beta_{2}\times {\mathrm{QFII}}_{i,t}+{\mathrm{Controls}}_{i,t}\\ \;+\mathrm{Year}+\mathrm{Industry}+\varepsilon \ldots \end{array}$$

(3) $$\begin{array}{l} {\mathrm{Innovation}}_{i,t}=\beta_{0}+\beta_{3}\times {\mathrm{QFII}}_{i,t}+\beta_{4}\times {\mathrm{RPTratio}}_{i,t}\\ /{\mathrm{Follow}}_{i,t}+{\mathrm{Controls}}_{i,t}+\mathrm{Year}+\mathrm{Industry}+\varepsilon \text{.} \end{array}$$

### Descriptive statistical analysis

Table 2A reports descriptive statistics for the main variables. The average QFIIdum of qualified foreign institutional investors is 0.103, indicating that 10.3% of the listed companies in China have qualified foreign institutional investors in their shareholder structures. The average value of QFIIshare is 0.108, indicating that the average shareholding ratio of qualified foreign institutional investors to the total shares of listed companies is 10.8%. The mean (standard deviation) of the total number of patent applications (Ln_Invtotal) is 1.155 (1.544), and the mean (standard deviation) of the patent applications for inventions (Ln_Invia) is 0.755 (1.191), which indicates that there are differences in innovation output and innovation quality among companies. After excluding specific items, the proportion of the sum of related party transactions in total assets (RPTratio) is 24.6% with a standard deviation of 0.352, and the sample distribution is similar to the sum of related party transactions. In terms of the number of analysts following, the mean of Follow is 1.452, and the standard deviation is 1.176, indicating that there are great differences in the degree of the number of analysts follow of different companies. The descriptive statistics of the remaining control variables are shown in Table 2A.

**Table 2A:** Descriptive statistics.

Panel A total sample descriptive statistics						
	N	Mean	Studded	Min	Median	Max
QFII	21,301	0.103	0.305	0	0	1
QFIIshare	21,301	0.108	0.411	0	0	2.59
Ln_Invtotal	21,301	1.155	1.544	0	0	5.911
Ln_Invia	21,301	0.755	1.191	0	0	5.081
RPTratio1	21,301	0.249	0.355	0	0.131	2.247
RPTratio2	21,301	0.246	0.352	0	0.129	2.21
Follow	21,301	1.452	1.176	0	1.386	3.664
Size	21,301	22.173	1.327	19.225	22.023	26.08
Lev	21,301	0.471	0.211	0.058	0.472	1.035
Roa	21,301	0.038	0.057	−0.207	0.035	0.215
Growth	21,301	0.069	0.315	−1.601	0.101	0.824
CFO	21,301	0.046	0.076	−0.199	0.046	0.261
Top1	21,301	36.178	15.355	8.77	34.42	76.69
Dual	21,301	0.202	0.402	0	0	1
Board	21,301	2.274	0.179	1.792	2.303	2.773
Indep	21,301	0.371	0.053	0.308	0.333	0.571
Soe	21,301	0.5	0.5	0	1	1
Age	21,301	2.777	0.366	1.386	2.833	3.434

Table 2B reports the results of the univariate difference test. This article is divided into two groups according to whether there are qualified foreign institutional investors (QFII), among which (1) and (2) are firms that do not have qualified foreign institutional investors, and (3) and (4) are listed as having qualified foreign institutional investment, and column (5) is the univariate difference between the two groups. From Table 2B, it can be seen that in a group with investment from overseas institutions, the mean value of Ln_Invtotal of corporate innovation is 1.462, and the mean value of Ln_Invia is 1.003; in a group without investment from overseas institutions, the mean value of Ln_Invtotal of corporate innovation is 1.119, and the mean value of Ln_Invia is 1.119. 0.726. This shows that when there are qualified foreign institutional investors, the level of corporate innovation is higher, which preliminarily verifies the research hypothesis of this paper.

**Table 2B:** Descriptive statistics grouped by QFII.

	(1)	(2)	(3)	(4)	(5)
Variables	QFII (0)	Mean1	QFII (1)	Mean2	MeanDiff
Ln_Invtotal	19097	1.119	2204	1.462	−0.343***
Ln_Invia	19097	0.726	2204	1.003	−0.277***
RPTratio2	19097	0.248	2204	0.222	0.026***
Follow	19097	1.367	2204	2.185	−0.818***

## Analysis of empirical results

### The impact of qualified foreign institutional investors (QFII) on corporate innovation

The empirical analysis of this paper is completed by STATA15. Table 3 reports the impact of QFII on the number of corporate innovations. Among them, the main explanatory variable of (1) and (2) is QFIIdum (If there is an institutional investor in the year of the firm, the value of this variable is 1, otherwise it is 0). Regression results of model (1) show that whether the control variable is added or not, the coefficient of QFIIdum is significantly positive, the regression coefficients of the explanatory variable QFIIdum are 0.369 and 0.184, and the significance level is both 1%, indicating that qualified foreign institutional investors can significantly increase the number of innovations of firms. In addition, the main explanatory variable in columns (3) and (4) of Table 3 is QFIIshare (the proportion of QFII shares in the total shares of listed companies). The results show that the regression coefficient of the explanatory variable QFIIshare is 0.258 without adding control variables, the coefficient drops to 0.154 after adding the control variable, but the significance level is still 1%, indicating that the shareholding ratio of qualified foreign institutional investors can significantly increase the number of innovations of firms. Therefore, the increase in the number of innovations in QFII in hypothesis 1 of this paper is supported. Further analysis of the regression coefficient of the control variable reveals that the regression coefficient of the company size variable Size, Roa, and CFO is significantly positive, and the regression coefficient of the asset-liability ratio variable Lev is significantly negative, indicating that compared with the company with smaller size, lower return on assets, less cash flow and higher leverage, the company’s innovation ability will be stronger than that of companies with smaller size, lower return on assets, less cash flow and higher leverage. In addition, the larger and younger the board, the higher the level of innovation.

**Table 3:** Qualified Foreign Institutional Investors (QFII) and the number of corporate innovations.

	(1)	(2)	(3)	(4)
	Ln_INVTotal	Ln_INVTotal	Ln_INVTotal	Ln_INVTotal
QFIIdum	0.369***	0.184***		
	(10.307)	(5.308)		
QFIIshare			0.258***	0.154***
			(9.217)	(5.726)
Size		0.212***		0.215***
		(21.224)		(21.498)
Lev		−0.295***		−0.302***
		(−5.931)		(−6.075)
Roa		1.559***		1.523***
		(8.766)		(8.579)
Growth		−0.047*		−0.047*
		(−1.871)		(−1.869)
CFO		0.854***		0.842***
		(7.127)		(7.037)
Top1		−0.001		−0.001
		(−1.380)		(−1.131)
Dual		0.007		0.006
		(0.280)		(0.240)
Board		0.245***		0.243***
		(3.722)		(3.688)
Indep		−0.023		−0.015
		(−0.106)		(−0.070)
Soe		−0.070***		−0.070***
		(−3.366)		(−3.335)
Age		−0.319***		−0.320***
		(−10.117)		(−10.158)
Constant	−0.025	−4.115***	−0.018	−4.160***
	(−0.412)	(−14.669)	(−0.301)	(−14.855)
Industry	Yes	Yes	Yes	Yes
Year	Yes	Yes	Yes	Yes
N	21,301	21,301	21,301	21,301
R-Square	0.231	0.271	0.231	0.272

*, **, *** are indicated to be significant at the 10, 5, and 1% levels, respectively, and the values in parentheses are *T*-values, as shown in the table below.

Table 4 reports the impact of QFII on the quality of corporate innovation. Among them, the main explanatory variable of (1) and (2) is QFIIdum. The results show that whether the control variable is added or not, the coefficient of QFIIdum is significantly positive, the regression coefficient of the explanatory variable QFIIdum is 0.305 and 0.145, and the significance level is 1%, indicating that the entry of qualified foreign institutional investors has significantly improved the innovation quality of firms. In addition, the main explanatory variable in columns (3) and (4) of Table 3 is QFIIshare. The results show that the regression coefficient of the explanatory variable QFIIshare is 0.212 without adding the control variable, and the coefficient after adding the control variable is 0.123, which is significant. The level is 1%, which also shows that the entry of qualified foreign institutional investors can significantly improve the innovation quality of firms. Therefore, the improvement of innovation quality by QFII in Hypothesis 1 of this paper is supported.

**Table 4:** Qualified Foreign Institutional Investors (QFII) and the quality of corporate innovation.

	(1)	(2)	(3)	(4)
	Ln_Invia	Ln_Invia	Ln_Invia	Ln_Invia
QFIIdum	0.305***	0.145***		
	(10.484)	(5.194)		
QFIIshare			0.212***	0.123***
			(9.389)	(5.756)
Size		0.185***		0.187***
		(22.308)		(22.535)
Lev		−0.170***		−0.175***
		(−4.396)		(−4.536)
Roa		1.231***		1.201***
		(8.824)		(8.636)
Growth		−0.036*		−0.036*
		(−1.897)		(−1.893)
CFO		0.550***		0.540***
		(6.001)		(5.903)
Top1		−0.002***		−0.002***
		(−3.780)		(−3.542)
Dual		0.026		0.025
		(1.302)		(1.263)
Board		0.217***		0.215***
		(4.118)		(4.083)
Indep		0.186		0.193
		(1.086)		(1.123)
Soe		0.028*		0.029*
		(1.674)		(1.706)
Age		−0.190***		−0.190***
		(−7.808)		(−7.850)
constant	−0.126***	−4.032***	−0.120***	−4.065***
	(−3.218)	(−17.282)	(−3.077)	(−17.456)
Industry	Yes	Yes	Yes	Yes
Year	Yes	Yes	Yes	Yes
*N*	21,301	21,301	21,301	21,301
*R*-square	0.184	0.229	0.184	0.230

### The impact of qualified foreign institutional investors (QFII) on corporate innovation—from the perspective of firms’ internal governance

Drawing on the research of Hou et al. (2017) and Jiang et al. (2020) on the “tunnel” behavior of firms, this paper uses hollowing-related transactions to measure the “tunnel” behavior of firms. Specifically, this paper measures the “tunnel” behavior of firms using the Sum of Annual Connected Transactions/Total Assets (RPTratio) metric that excludes five types of transaction categories, namely collaborative projects, licensing agreements, research and development results, key management compensation, and other matters. Table 5A reports the internal governance mechanism of QFII to increase the number of corporate innovations by reducing the insider “tunnel” of holding companies. Among them, the main explanatory variable of (1) and (2) is QFIIdum, and the main explanatory variable of columns (3) and (4) is QFIIshare. Specifically, the regression results of model (1) in Table 3 show that the regression coefficient of QFIIdum is 0.184, which is significantly positive at the 1% level. Further, combined with models (2) and (3), it is found (see Table 5A for details) that the regression coefficient of QFIIdum to RPTratio is-0.018 (significant at the 1% level), indicating that QFIIdum can significantly reduce the “tunnel” behaviors in firms; simultaneously regressing QFIIdum and RPTratio on INVTotal, we found that the regression coefficient of RPTratio was-0.241 (significant at the 1% level). More importantly, the regression coefficient of QFIIdum decreased to 0.18, and the significance level did not change, indicating that RPTratio played a partial mediating effect, that is, QFIIdum can increase the number of innovations of firms by reducing insiders’ “tunnel” of holding companies. Columns (3) and (4) are similar to columns (1) and (2), under the influence of RPTratio, the regression coefficient of QFIIshare decreased from 0.154 to 0.151, and the significance level did not change, indicating that RPTratio played a part in mediating effect. To sum up, we found that QFII can improve the number of innovations of firms by improving the behaviors of major shareholders in China.

**Table 5A:** The impact of QFII on the number of innovations: A mechanism test based on the behavior of the firm’s “tunnel.”

	(1)	(2)	(3)	(4)
	RPTratio	INVTotal	RPTratio	INVTotal
RPTTratio		−0.241***		−0.241***
		(−8.398)		(−8.404)
QFIIdum	−0.018***	0.180***		
	(−2.608)	(5.183)		
QFIIshare			−0.011**	0.151***
			(−2.155)	(5.631)
Size	−0.023***	0.207***	−0.023***	0.209***
	(−8.385)	(20.564)	(−8.566)	(20.817)
Lev	0.494***	−0.176***	0.495***	−0.183***
	(25.412)	(−3.412)	(25.488)	(−3.544)
Roa	−0.160**	1.521***	−0.159**	1.485***
	(−2.227)	(8.562)	(−2.212)	(8.375)
Growth	−0.027***	−0.054**	−0.027***	−0.054**
	(−2.652)	(−2.143)	(−2.649)	(−2.141)
CFO	0.004	0.855***	0.004	0.843***
	(0.098)	(7.160)	(0.093)	(7.069)
Top1	0.003***	−0.000	0.003***	−0.000
	(17.687)	(−0.298)	(17.618)	(−0.053)
Dual	−0.018***	0.003	−0.018***	0.002
	(−3.036)	(0.108)	(−3.034)	(0.067)
Board	0.004	0.246***	0.004	0.244***
	(0.237)	(3.743)	(0.244)	(3.710)
Indep	−0.126***	−0.053	−0.127***	−0.046
	(−2.625)	(−0.249)	(−2.639)	(−0.214)
Soe	0.024***	−0.064***	0.024***	−0.064***
	(4.392)	(−3.092)	(4.373)	(−3.062)
Age	0.086***	−0.298***	0.086***	−0.299***
	(12.184)	(−9.436)	(12.180)	(−9.478)
Constant	0.209***	−4.064***	0.217***	−4.107***
	(3.241)	(−14.522)	(3.373)	(−14.699)
Industry	Yes	Yes	Yes	Yes
Year	Yes	Yes	Yes	Yes
*N*	21,301	21,301	21,301	21,301
*R*-square	0.116	0.274	0.116	0.274

Table 5B reports the internal governance mechanism of QFII to improve the quality of corporate innovation by reducing the insiders’ “tunnel” of holding companies. As in Table 5A, the main explanatory variable in (1) and (2) is QFIIdum, and the main explanatory variable in columns (3) and (4) is QFIIshare. Specifically, the regression results of model (1) in Table 3 show that the regression coefficient of QFII is 0.145, which is significantly positive at the 1% level. Further, combined with models (2) and (3), it is found (see Table 5B for details) that the regression coefficient of QFII on RPTratio is-0.018 (significant at the 1% level), indicating that QFIIdum can significantly reduce the behaviors of “tunnel” of firms’; simultaneously regressing QFIIdum and RPTratio on INVTotal, we found that the regression coefficient of RPTratio was-0.18 (significant at the 1% level). More importantly, the regression coefficient of QFIIdum dropped to 0.121, and the significance level did not change, indicating that RPTratio played a partial mediating effect, that is, QFII can improve the innovation quality of firms by reducing insiders’ “tunnel” of holding companies. Columns (3) and (4) are similar to columns (1) and (2), under the influence of RPTratio, the regression coefficient of QFIIshare decreased from 0.123 to 0.121, and the significance level did not change, indicating that RPTratio played a part in mediating effect. To sum up, we found that QFII can improve the innovation quality of firms by improving the behavior of major shareholders in China.

**Table 5B:** The impact of QFII on innovation quality: Mechanism test based on the firm’s “tunnel” behavior.

	(1)	(2)	(3)	(4)
	RPTTratio	Ln_Invia	RPTTratio	Ln_Invia
RPTTratio		−0.180***		−0.180***
		(−8.245)		(−8.247)
QFIIdum	−0.018***	0.142***		
	(−2.608)	(5.079)		
QFIIshare			−0.011**	0.121***
			(−2.155)	(5.672)
Size	−0.023***	0.181***	−0.023***	0.183***
	(−8.385)	(21.731)	(−8.566)	(21.939)
Lev	0.494***	−0.081**	0.495***	−0.086**
	(25.412)	(−2.007)	(25.488)	(−2.137)
Roa	−0.160**	1.202***	−0.159**	1.172***
	(−2.227)	(8.624)	(−2.212)	(8.436)
Growth	−0.027***	−0.041**	−0.027***	−0.041**
	(−2.652)	(−2.163)	(−2.649)	(−2.158)
CFO	0.004	0.551***	0.004	0.541***
	(0.098)	(6.028)	(0.093)	(5.930)
Top1	0.003***	−0.001***	0.003***	−0.001**
	(17.687)	(−2.785)	(17.618)	(−2.550)
Dual	−0.018***	0.022	−0.018***	0.021
	(−3.036)	(1.143)	(−3.034)	(1.104)
Board	0.004	0.218***	0.004	0.216***
	(0.237)	(4.138)	(0.244)	(4.103)
Indep	−0.126***	0.164	−0.127***	0.170
	(−2.625)	(0.955)	(−2.639)	(0.991)
Soe	0.024***	0.032*	0.024***	0.033**
	(4.392)	(1.939)	(4.373)	(1.970)
Age	0.086***	−0.174***	0.086***	−0.175***
	(12.184)	(−7.159)	(12.180)	(−7.202)
Constant	0.209***	−3.994***	0.217***	−4.026***
	(3.241)	(−17.159)	(3.373)	(−17.325)
Industry	Yes	Yes	Yes	Yes
Year	Yes	Yes	Yes	Yes
*N*	21,301	21,301	21,301	21,301
*R*-square	0.116	0.232	0.116	0.232

### The impact of qualified foreign institutional investors (QFII) on corporate innovation—from the perspective of external analysts

In terms of the measurement of analysts following, the mediating variable Follow is the number of analysts following. Referring to the existing literature practice (Chen et al., 2017), this variable is measured using the natural logarithm of the number of analysts who publish surplus forecasts for a company in a given year plus 1. When the surplus forecast is released by the analyst team, the number of trackers is considered 1 person. Based on this measurement method, we examined the mediating effects of analysts following. The specific results are as follows: Table 6A reports on the internal governance mechanisms of QFII to increase the number of corporate innovations by attracting more analysts following Similar to the test mechanism in Table 5A, based on the regression coefficient of QFIIdum shown in model (1) being significantly positive, considering model (2) and (3) we found (the empirical results are detailed in Table 6A) the regression coefficient of QFIIdum to Follow was 0.386 (significant at the level of 1%), indicating that QFIIdum can significantly reduce the behaviors of “tunnel” of firms; Returning QFIIdum and Follow to INVTotal at the same time revealed that Follow’s regression coefficient was 0.187 (significant at the 1% level). More importantly, QFIIdum’s regression coefficient dropped to 0.112, and the significance level did not change, indicating that Follow had a partial mediating effect, that is, QFIIdum was able to increase the number of innovations in the firm by reducing the insiders’ “tunnel” of the holding company. Columns (3) and (4) are similar to columns (1) and (2), and under the influence of RPTratio, the regression coefficient of QFIIshare decreased from 0.154 to 0.096, and the significance level did not change, indicating that Follow played a part of the mediating role. Taken together, we found that QFII can improve the number of innovations in a business by attracting more analysts following.

**Table 6A:** The impact of QFII on the number of innovations: a mechanism test based on the number of analysts followed.

	(1)	(2)	(3)	(4)
	Follow	INVTotal	Follow	INVTotal
Follow		0.187***		0.186***
		(18.009)		(17.851)
QFIIdum	0.386***	0.112***		
	(18.955)	(3.224)		
QFIIshare			0.312***	0.096***
			(20.386)	(3.550)
Size	0.475	0.123***	0.480	0.125***
	(81.634)	(11.097)	(83.614)	(11.263)
Lev	−0.544***	−0.193***	−0.560***	−0.198***
	(−14.522)	(−3.888)	(−14.974)	(−3.989)
Roa	5.299***	0.567***	5.231***	0.551***
	(37.092)	(3.181)	(36.656)	(3.096)
Growth	0.108***	−0.068***	0.108***	−0.067***
	(5.026)	(−2.740)	(5.065)	(−2.731)
CFO	0.921***	0.681***	0.901***	0.675***
	(10.143)	(5.717)	(9.929)	(5.666)
Top1	−0.003***	−0.000	−0.003***	−0.000
	(−7.710)	(−0.393)	(−6.959)	(−0.246)
Dual	0.100***	−0.012	0.098***	−0.012
	(6.238)	(−0.480)	(6.124)	(−0.503)
Board	0.269***	0.195***	0.265***	0.194***
	(6.593)	(2.971)	(6.492)	(2.955)
Indep	0.070	−0.036	0.086	−0.031
	(0.524)	(−0.169)	(0.647)	(−0.146)
Soe	−0.208***	−0.031	−0.206***	−0.031
	(−14.712)	(−1.511)	(−14.605)	(−1.506)
Age	−0.446***	−0.235***	−0.447***	−0.237***
	(−21.850)	(−7.400)	(−21.908)	(−7.450)
constant	−8.538	−2.516***	−8.641	−2.553***
	(−52.447)	(−8.556)	(−53.450)	(−8.689)
Industry	Yes	Yes	Yes	Yes
Year	Yes	Yes	Yes	Yes
N	21,301	21,301	21,301	21,301
R-Square	0.442	0.283	0.444	0.283

Table 6B reports on the internal governance mechanisms of QFII to increase the number of innovations in firms by attracting more analysts to follow. Similar to the test mechanism in Table 6A, on the basis that the regression coefficient of QFIIdum shown in model (1) is significantly positive, considering models (2) and (3) we found, (the empirical results are detailed in Table 6B), that the regression coefficient of QFIIdum to Follow is 0.386 (significant at the level of 1%), that is, QFIIdum can significantly reduce the behaviors of “tunnel” of firms; Returning QFIIdum and Follow to INVTotal at the same time revealed that Follow’s regression coefficient was 0.138 (significant at the 1% level). More importantly, QFIIdum’s regression coefficient dropped to 0.092, and the significance level did not change, indicating that Follow played a partial mediation effect, that is, QFIIdum can improve the quality of innovation by reducing insiders’ “tunnel” of the holding company. Similar to columns (1) and (2), QFIIshare’s regression coefficient decreased from 0.123 to 0.081 under the influence of RPTratio, and the significance level did not change, indicating that Follow played a part in the mediating role. Taken together, we found that QFII can improve the quality of innovation in a business by attracting more analysts to follow.

**Table 6B:** The impact of QFII on innovation quality: a mechanism test based on analysts following quantity.

	(2)	(3)	(5)	(6)
	Follow	Ln_Invia	Follow	Ln_Invia
Follow		0.138***		0.137***
		(16.821)		(16.623)
QFIIdum	0.386***	0.092***		
	(18.955)	(3.273)		
QFIIshare			0.312***	0.081***
			(20.386)	(3.719)
Size	0.475	0.120***	0.480	0.121***
	(81.634)	(13.004)	(83.614)	(13.152)
Lev	−0.544***	−0.095**	−0.560***	−0.099**
	(−14.522)	(−2.447)	(−14.974)	(−2.551)
Roa	5.299***	0.499***	5.231***	0.486***
	(37.092)	(3.580)	(36.656)	(3.489)
Growth	0.108***	−0.051***	0.108***	−0.051***
	(5.026)	(−2.737)	(5.065)	(−2.725)
CFO	0.921***	0.423***	0.901***	0.417***
	(10.143)	(4.626)	(9.929)	(4.567)
Top1	−0.003***	−0.002***	−0.003***	−0.001***
	(−7.710)	(−2.893)	(−6.959)	(−2.749)
Dual	0.100***	0.012	0.098***	0.011
	(6.238)	(0.60 3)	(6.124)	(0.580)
Board	0.269***	0.180***	0.265***	0.179***
	(6.593)	(3.431)	(6.492)	(3.415)
Indep	0.070	0.177	0.086	0.181
	(0.524)	(1.037)	(0.647)	(1.062)
Soe	−0.208***	0.057***	−0.206***	0.057***
	(−14.712)	(3.409)	(−14.605)	(3.414)
Age	−0.446***	−0.128***	−0.447***	−0.129***
	(−21.850)	(−5.213)	(−21.908)	(−5.267)
constant	−8.538	−2.853***	−8.641	−2.884***
	(−52.447)	(−11.751)	(−53.450)	(−11.880)
Industry	Yes	Yes	Yes	Yes
Year	Yes	Yes	Yes	Yes
*N*	21,301	21,301	21,301	21,301
*R*-square	0.442	0.239	0.444	0.240

### Robustness check

#### Solve endogenous problems

First, we used the test based on Propensity Score Matching (PSM). The basic assumption of the test is that the explanatory variables are exogenous, but the investment of QFII in the firm may be an endogenous decision to a large extent, which will be affected by the characteristics of the company, so in the robustness test, this paper uses the PSM method to control the potential endogenous problems. Since PSM requires that a certain matching standard be chosen, this paper includes the existing influencing factors affecting the investment choice of QFII into the logical model, and the specific matching variables are firm leverage (Lev), firm size (Size), Profitability (Roa), Growth, Cash Flow (CFO), and Age of Business. In addition, we also added the mean value of provincial QFII input as a matching variable to reduce the endogeneity problem in QFII selection. On this basis, we did a 1-to-1 replaceable match for each sample of firms with QFII investments. In the test of the pairing effect, we found that the post-matching PSM’s common support hypothesis and equilibrium hypothesis were satisfied. After controlling for the potential endogeneity in the process of QFII investing in firms, the conclusion in this paper’s hypothesis still holds. The specific test results are shown in Table 7.

**Table 7:** Robustness test: The impact of QFII on the quantity and quality of corporate innovation—a PSM pair-based test.

	(1)	(2)	(3)	(4)
	Ln_INVTotal	Ln_INVTotal	Ln_Invia	Ln_Invia
QFIIdum	0.158***		0.124***	
	(3.208)		(3.221)	
QFIIshare		0.127***		0.103***
		(3.832)		(4.032)
Size	0.334***	0.339***	0.269***	0.274***
	(12.414)	(12.587)	(12.927)	(13.105)
Lev	−0.436***	−0.457***	−0.325***	−0.342***
	(−2.899)	(−3.044)	(−2.756)	(−2.904)
Roa	2.678***	2.525***	1.866***	1.742***
	(5.073)	(4.814)	(4.421)	(4.148)
Growth	−0.075	−0.075	−0.036	−0.036
	(−0.884)	(−0.895)	(−0.552)	(−0.561)
CFO	0.538	0.473	0.337	0.285
	(1.604)	(1.414)	(1.305)	(1.108)
Top1	−0.002	−0.002	−0.003**	−0.002*
	(−1.432)	(−1.091)	(−1.990)	(−1.648)
Dual	0.157**	0.153**	0.087	0.084
	(2.247)	(2.208)	(1.599)	(1.555)
Board	0.620***	0.607***	0.544***	0.534***
	(3.719)	(3.638)	(4.252)	(4.168)
Indep	0.420	0.432	0.814*	0.824*
	(0.770)	(0.793)	(1.873)	(1.901)
Soe	−0.204***	−0.202***	−0.097**	−0.095**
	(−3.503)	(−3.473)	(−2.132)	(−2.102)
Age	−0.040	−0.042	−0.012	−0.015
	(−0.473)	(−0.501)	(−0.191)	(−0.226)
Constant	−8.097***	−8.182***	−6.978***	−7.048***
	(−11.385)	(−11.522)	(−12.586)	(−12.733)
Industry	Yes	Yes	Yes	Yes
Year	Yes	Yes	Yes	Yes
N	3,918	3,918	3,918	3,918
R-Square	0.320	0.321	0.286	0.288

Second, we adopted the Heckman two-stage regression. Since the governance characteristics of firms themselves may lead QFII to choose such firms for investment, we divided the samples into two groups according to whether they have QFII investment or not. Then, the Heckman two-stage method is adopted to solve the self-selection problem in the samples. In the first stage, we used the average QFII_IV1 of the QFIIshares of other companies in the same industry as the company and the average QFII_IV2 of the QFIIshares of other companies in the same province as the instrumental variables. In the second stage, we put into the regression model The Inverse Mills Ratio (IMR) estimated in the first stage. Columns (2) to (6) of Table 8 report the regression results in the second stage. It can be seen that after controlling the problem of endogenous selection bias, the research conclusions are consistent with the above.

**Table 8:** Robustness test: The impact of QFII on the quantity and quality of corporate innovation—based on Heckman’s test.

	(1)	(2)	(3)	(4)	(5)
	QFII	Ln_INVTotal	Ln_Invia	Ln_INVTotal	Ln_Invia
QFII_IV1	6.004***				
	(6.831)				
QFII_IV1	16.529***				
	(4.070)				
QFIIdum		0.178***	0.140***		
		(5.101)	(4.996)		
QFIIshare				0.147***	0.118***
				(5.443)	(5.449)
IMR		−0.464***	−0.416***	−0.459***	−0.413***
		(−8.120)	(−9.349)	(−8.042)	(−9.263)
Size	0.458***	0.019	0.012	0.023	0.015
	(20.035)	(0.698)	(0.550)	(0.851)	(0.703)
Lev	−0.869***	0.088	0.172***	0.078	0.164***
	(−5.576)	(1.266)	(3.184)	(1.114)	(3.027)
Roa	4.696***	−0.415	−0.554**	−0.430	−0.567**
	(7.503)	(−1.348)	(−2.304)	(−1.400)	(−2.359)
Growth	0.023	−0.065**	−0.051***	−0.065**	−0.051***
	(0.263)	(−2.553)	(−2.648)	(−2.544)	(−2.636)
CFO	2.243***	−0.082	−0.284**	−0.084	−0.286**
	(5.971)	(−0.491)	(−2.196)	(−0.505)	(−2.214)
Top1	0.005***	−0.003***	−0.004***	−0.003***	−0.004***
	(3.256)	(−4.412)	(−6.929)	(−4.165)	(−6.694)
Dual	0.156**	−0.063**	−0.036*	−0.064**	−0.036*
	(2.479)	(−2.423)	(−1.747)	(−2.439)	(−1.764)
Board	0.021	0.233***	0.205***	0.231***	0.203***
	(0.133)	(3.524)	(3.880)	(3.492)	(3.847)
Indep	−0.204	0.041	0.252	0.048	0.257
	(−0.414)	(0.191)	(1.463)	(0.222)	(1.494)
Soe	0.169***	−0.148***	−0.041**	−0.146***	−0.040**
	(2.966)	(−6.532)	(−2.268)	(−6.472)	(−2.208)
Age	0.408***	−0.475***	−0.332***	−0.475***	−0.331***
	(5.067)	(−12.636)	(−11.405)	(−12.620)	(−11.387)
constant	−15.166***	1.495**	0.996*	1.397*	0.919
	(−20.317)	(1.964)	(1.664)	(1.836)	(1.534)
Industry	Yes	Yes	Yes	Yes	Yes
Year	Yes	Yes	Yes	Yes	Yes
*N*	21,122	21,122	21,122	21,122	21,122
Pr/*R* square	0.090	0.274	0.232	0.274	0.233

#### Other robustness checks

First, R&D investment (Ln_Rdexp: the natural logarithm of R&D expenditure plus 1) and the number of utility model applications (Ln_Umia: the natural logarithm of the number of utility model patent applications plus 1) are used to measure the level of corporate innovation. In the regression results in Table 9, the regression coefficients of QFIIdum and QFIIshare for both types of innovation variables are significantly positive, indicating that qualified foreign institutional investors can promote the improvement of corporate innovation levels. The research conclusions are consistent with the above.

**Table 9:** Robustness test: the impact of QFII on corporate innovation under changing the measurement method of innovation level.

	(1)	(2)	(3)	(4)
	Ln_RDexp	Ln_RDexp	Ln_Umia	Ln_Umia
QFIIdum	0.100***		0.126***	
	(2.822)		(4.413)	
QFIIshare		0.102***		0.105***
		(4.098)		(4.676)
Size	0.829	0.829	0.159***	0.161***
	(73.665)	(74.023)	(19.284)	(19.479)
Lev	−0.566***	−0.570***	−0.096**	−0.101**
	(−8.249)	(−8.305)	(−2.430)	(−2.556)
Roa	3.378***	3.345***	0.822***	0.798***
	(13.285)	(13.142)	(5.871)	(5.715)
Growth	0.143***	0.145***	−0.059***	−0.059***
	(2.957)	(2.983)	(−2.899)	(−2.897)
CFO	0.949***	0.943***	0.531***	0.523***
	(5.064)	(5.031)	(5.502)	(5.428)
Top1	−0.002***	−0.002**	0.000	0.000
	(−2.613)	(−2.504)	(0.374)	(0.586)
Dual	0.101***	0.100***	−0.038*	−0.039**
	(4.519)	(4.486)	(−1.958)	(−1.996)
Board	0.034	0.033	0.181***	0.180***
	(0.437)	(0.426)	(3.400)	(3.370)
Indep	0.318	0.328	0.056	0.061
	(1.419)	(1.463)	(0.326)	(0.356)
Soe	−0.092***	−0.091***	−0.085***	−0.084***
	(−3.626)	(−3.589)	(−5.047)	(−5.022)
Age	−0.338***	−0.339***	−0.211***	−0.211***
	(−11.235)	(−11.268)	(−8.242)	(−8.278)
Constant	−2.476***	−2.492***	−3.363***	−3.394***
	(−7.652)	(−7.712)	(−14.531)	(−14.683)
Industry	Yes	Yes	Yes	Yes
Year	Yes	Yes	Yes	Yes
*N*	14,022	14,022	21,135	21,135
*R*-square	0.538	0.539	0.263	0.263

Second, to solve the problem of missing variables or reverse causality, this paper lags the independent variables (QFIIdum, QFIIshare) by one period. The regression results in Table 10 show that the regression coefficients of L.QFIIdum and L.QFIIshare are both significantly positive at the 1% level, and the research conclusions remain unchanged.

**Table 10:** Robustness test: the impact of QFII on corporate innovation with one period lag.

	(1)	(2)	(3)	(4)
	Ln_INVTotal	Ln_INVTotal	Ln_Invia	Ln_Invia
L.QFIIdum	0.194***		0.145***	
	(5.080)		(4.874)	
L.QFIIshare		0.182***		0.130***
		(5.952)		(5.541)
Size	0.236***	0.237***	0.191***	0.193***
	(20.304)	(20.516)	(21.130)	(21.353)
Lev	−0.277***	−0.282***	−0.155***	−0.159***
	(−4.925)	(−5.024)	(−3.583)	(−3.686)
Roa	1.562***	1.506***	1.268***	1.231***
	(7.807)	(7.560)	(8.129)	(7.920)
Growth	−0.042	−0.043	−0.030	−0.030
	(−1.516)	(−1.538)	(−1.421)	(−1.451)
CFO	0.915***	0.896***	0.573***	0.560***
	(6.756)	(6.619)	(5.614)	(5.492)
Top1	−0.002**	−0.001*	−0.002***	−0.002***
	(−2.179)	(−1.952)	(−3.992)	(−3.782)
Dual	−0.013	−0.015	0.010	0.008
	(−0.453)	(−0.535)	(0.440)	(0.373)
Board	0.263***	0.261***	0.218***	0.217***
	(3.501)	(3.475)	(3.755)	(3.732)
Indep	−0.013	−0.007	0.203	0.208
	(−0.054)	(−0.030)	(1.088)	(1.115)
Soe	−0.063***	−0.063***	0.037**	0.037**
	(−2.708)	(−2.695)	(2.022)	(2.044)
Age	−0.314***	−0.316***	−0.196***	−0.197***
	(−8.507)	(−8.587)	(−6.926)	(−6.983)
Constant	−4.516***	−4.547***	−4.067***	−4.096***
	(−13.744)	(−13.878)	(−15.838)	(−15.988)
Industry	Yes	Yes	Yes	Yes
Year	Yes	Yes	Yes	Yes
*N*	18,404	18,404	18,404	18,404
*R*-square	0.271	0.272	0.232	0.232

## Further analysis: QFII shareholding, corporate innovation, and corporate efficiency

In the context of economic globalization, the opening up of emerging capital markets is an inevitable trend of development. One of the significances of China’s implementation of the QFII system is to cultivate strategic institutional investors, improve the governance structure of listed companies, and improve the quality of listed companies. In the past 20 years of development, the scale of the QFII market has been growing. However, the academic community has not reached an agreement on whether QFII shareholding can improve the efficiency of enterprises. Some scholars are optimistic about the “value creation” ability of QFII. They believe that QFII shares are highly specialized and independent, and can actively participate in corporate governance and effectively supervise the management or shareholders (Ferreira and Matos, 2008; Elyasiani et al., 2010; Aggarwal et al., 2011), which can improve enterprise efficiency (Ferreira and Matos, 2008; Aggarwal et al., 2011); Other scholars believe that QFII is just a “value investor.” They chose excellent companies to invest in but did not make substantial contributions to the improvement of enterprise efficiency (Fu, 2008; Li and Han, 2014). So, does QFII play a role as a value creator to actively improve the performance of listed companies in China’s capital market?

Based on the above empirical results in this paper, we have found that QFII with value investment can play a corporate governance role by effectively curbing major shareholders’ “tunnel” of a firm; by attracting more analysts to follow, it can attract common attention to the innovation activities of firms under the role of signal transmission. QFII has a positive impact on the quantity and quality of innovation in firms in terms of improving the internal and external environment of firms. The improvement of the quantity and quality of innovation will usually have a positive impact on the value of the firm, so in further analysis, we explored whether the QFII system can improve the efficiency of the firm by promoting corporate innovation. To this end, we examined the impact of QFII shareholding on the performance and value of firms focusing on how QFII promotes the output and quality of corporate innovation. Table 11 reports the regression results of the following periods of firm performance (ROA) and firm value (TobinQ) as interpreted variables, and the regression results show that the regression coefficients of QFIIdum*Ntotal and QFIIdum*Nvia are significantly positive (The coefficient of QFIIdum*Nvia for ROA _t + 1_ regression is not significant, indicating that the impact of QFII on firm performance through innovation needs to be improved), and overall, The empirical results of Table 11 show that the increase in innovation output and the improvement of innovation quality brought about by QFII shareholding can increase the future performance of firms and improve firm value to a certain extent, that is, QFII plays a role as a value creator who actively improves the efficiency of listed companies in China’s capital market.

**Table 11:** QFII, corporate innovation, corporate performance, and value.

	(1)	(2)	(3)	(4)	(5)	(6)	(7)	(8)
	Whether QFII holds shares				QFII shareholding ratio			
	ROA_t + 1_	ROA_t + 1_	TobinQ_t + 1_	TobinQ_t + 1_	ROA_t + 1_	ROA_t + 1_	TobinQ_t + 1_	TobinQ_t + 1_
Ln_INVTotal	0.001***		−0.020***		0.001***		−0.017***	
	(4.702)		(−3.641)		(4.737)		(−3.170)	
QFIIdum*Ntotal	0.001**		0.090***		0.001***		0.062***	
	(2.322)		(6.712)		(2.976)		(6.261)	
Ln_Invia		0.002***		0.002		0.001***		0.005
		(5.220)		(0.320)		(5.074)		(0.717)
QFIIdum*Nvia		0.000		0.088***		0.001		0.064***
		(0.633)		(5.142)		(1.579)		(4.884)
QFIIdum	0.007***	0.008***	0.064**	0.102***	0.006***	0.007***	0.035	0.060***
	(5.811)	(7.129)	(2.135)	(3.480)	(6.536)	(7.533)	(1.539)	(2.675)
Size	−0.000	−0.000	−0.486	−0.490	−0.000	−0.000	−0.482	−0.487
	(−0.570)	(−0.599)	(−44.901)	(−45.050)	(−0.351)	(−0.386)	(−44.879)	(−45.035)
Lev	−0.025***	−0.025***	0.178**	0.184***	−0.026***	−0.026***	0.165**	0.172**
	(−7.640)	(−7.653)	(2.569)	(2.655)	(−7.763)	(−7.770)	(2.385)	(2.477)
Roa	0.395***	0.395***	2.619***	2.600***	0.393***	0.393***	2.584***	2.566***
	(28.946)	(28.962)	(9.700)	(9.631)	(28.757)	(28.780)	(9.534)	(9.470)
Growth	0.007***	0.006***	−0.177***	−0.177***	0.007***	0.006***	−0.178***	−0.177***
	(3.524)	(3.505)	(−4.840)	(−4.831)	(3.527)	(3.515)	(−4.865)	(−4.843)
CFO	0.123***	0.124***	0.908***	0.896***	0.123***	0.123***	0.909***	0.893***
	(19.177)	(19.222)	(6.478)	(6.382)	(19.068)	(19.106)	(6.474)	(6.360)
Top1	0.000***	0.000***	−0.000	−0.000	0.000***	0.000***	−0.000	−0.000
	(8.208)	(8.293)	(−0.635)	(−0.550)	(8.574)	(8.650)	(−0.252)	(−0.166)
Dual	−0.000	−0.000	0.037*	0.038*	−0.000	−0.000	0.035	0.035
	(−0.104)	(−0.112)	(1.716)	(1.748)	(−0.200)	(−0.203)	(1.616)	(1.627)
Board	0.005**	0.005**	0.022	0.015	0.005**	0.004**	0.022	0.014
	(2.045)	(2.007)	(0.426)	(0.289)	(2.015)	(1.973)	(0.427)	(0.279)
Indep	−0.005	−0.005	1.520***	1.516***	−0.005	−0.005	1.536***	1.528***
	(−0.703)	(−0.750)	(8.945)	(8.912)	(−0.640)	(−0.696)	(9.026)	(8.972)
Soe	−0.004***	−0.004***	0.012	0.012	−0.004***	−0.004***	0.013	0.014
	(−5.232)	(−5.425)	(0.664)	(0.685)	(−5.195)	(−5.379)	(0.737)	(0.752)
Age	−0.000	−0.000	0.222***	0.230***	−0.000	−0.000	0.222***	0.230***
	(−0.295)	(−0.336)	(9.267)	(9.570)	(−0.390)	(−0.432)	(9.256)	(9.559)
constant	−0.003	−0.002	10.664	10.746	−0.005	−0.004	10.581	10.669
	(−0.304)	(−0.224)	(46.142)	(46.223)	(−0.495)	(−0.401)	(46.067)	(46.158)
Industry	Yes	Yes	Yes	Yes	Yes	Yes	Yes	Yes
Year	Yes	Yes	Yes	Yes	Yes	Yes	Yes	Yes
*N*	21,259	21,259	20,703	20,703	21,259	21,259	20,703	20,703
*R*-square	0.307	0.307	0.355	0.354	0.308	0.308	0.355	0.354

## Research conclusion

Innovation is the main driving force for long-term economic development. Therefore, how to transform from “high-volume and low-quality” to “high-volume and high-quality” is an important issue in China’s transformation and development at the present stage. From the perspective of introducing qualified foreign institutional investors and using the data of China’s A-share listed companies from 2007 to 2018. The research finds that: (1) QFII shareholding has significantly improved the innovation level of the invested firms, which is manifested in the increase in innovation output and the improvement of innovation quality. (2) QFII shareholding can improve the level of corporate innovation by slowing down insiders’ “tunnel” of the holding company and increasing the number of analysts following, which indicates that QFII can improve the governance structure of the listed companies and improve their quality. Through further research, we found that QFII shareholding significantly improved corporate efficiency by enhancing the level of corporate innovation, indicating that QFII can play the role of “value creators” that improve corporate efficiency.

The research results of this paper show that China’s QFII system has achieved phased results, and the introduction of high-quality investors in the capital market will not only help improve the internal and external governance structure of firms but also improve the efficiency of firms by promoting the level of corporate innovation, thereby promoting the high-quality development of China’s real economy. The conclusions of the above research have certain practical guiding significance for Chinese corporate innovation, governance structure and effective operation of the capital market: (1) For listed companies, when firms need to introduce foreign capital, the introduction of QFII investment is an effective financing channel, which can not only bring corresponding financial support to firms, but also improve the internal and external governance environment of firms and promote the improvement of the quantity and quality of corporate innovation; (2) from the perspective of corporate governance, firms need to actively integrate with the world, and the introduction of QFII investment is conducive to improving the internal governance environment of firms and reducing internal “tunnel”; at the same time, it is also conducive to driving the number of external analysts to follow, drawing attention to firms, and providing favorable environmental support for corporate innovation; (3) as far as the construction of China’s capital market is concerned, QFII provides support for the construction of a mature and efficient capital market, which is in line with an important part of Chinese traditional wisdom— opening up, cultural inclusiveness and win-win cooperation. The continuous exploration and opening up of the capital market provide important support for the continuous innovation, transformation, and upgrading of China’s entity firms. China should continue to adhere to the reasonable and orderly policy of opening up, building a diversified and stable capital market, and providing an open and stable capital market for the high-quality development of firms.

There are still some deficiencies in this paper to be studied in the future: (1) The selection of corporate innovation indicators has limitations and does not reflect the effectiveness of the variable design. Comprehensive indicators can be expected to try in future research. (2) This paper uses the data on corporate innovation nationwide and does not divide the different regions where the enterprises are located, which may affect the results to some extent.

## Data availability statement

Publicly available datasets were analyzed in this study. This data can be found at: CSMAR: www.csmar.com; CNRD: www.cnrds.com.

## Author contributions

XW: conceptualization, methodology, software, investigation, formal analysis, and writing–original draft. WW: conceptualization, resources, supervision, and writing–review and editing. XS: software and validation. All authors contributed to the article and approved the submitted version.

## Conflict of interest

The authors declare that the research was conducted in the absence of any commercial or financial relationships that could be construed as a potential conflict of interest.

## Publisher’s note

All claims expressed in this article are solely those of the authors and do not necessarily represent those of their affiliated organizations, or those of the publisher, the editors and the reviewers. Any product that may be evaluated in this article, or claim that may be made by its manufacturer, is not guaranteed or endorsed by the publisher.

## References

[ref1] Admati A. R. Pfleiderer P. (2009). The “wall street walk” and shareholder activism: exit as a form of voice. Rev. Financ. Stud. 22, 2645–2685. doi: 10.1093/rfs/hhp037

[ref2] Aggarwal R. Erel I. Ferreira M. Matos P. (2011). Does governance travel around the world? Evidence from institutional investors. J. Financ. Econ. 100, 154–181. doi: 10.1016/j.jfineco.2010.10.018

[ref3] Aghion P. Van Reenen J. Zingales L. (2013). Innovation and institutional ownership. Am. Econ. Rev. 103, 277–304. doi: 10.1257/aer.103.1.277

[ref4] Ain Q. U. Yuan X. Javaid H. M. (2021). The impact of board gender diversity and foreign institutional investors on firm innovation: evidence from China. Eur. J. Innov. Manag.

[ref5] Anwar S. Sun S. (2014). Heterogeneity and curvilinearity of FDI-related productivity spillovers in China's manufacturing sector. Econ. Model. 41, 23–32. doi: 10.1016/j.econmod.2014.03.021

[ref6] Areneke G. Adegbite E. Tunyi A. (2022). Transfer of corporate governance practices into weak emerging market environments by foreign institutional investors. Int. Bus. Rev. 31:101978. doi: 10.1016/j.ibusrev.2022.101978

[ref8] Baruffaldi S. H. Simeth M. Wehrheim D. (2022). Asymmetric information and R&D disclosure: evidence from scientific publications. Unpublished working paper, University of Bath, Copenhagen Business School, and IESE Business School.

[ref9] Bekaert G. Harvey C. R. (2000). Foreign speculators and emerging equity markets. J. Financ. 55, 565–613. doi: 10.1111/0022-1082.00220

[ref10] Bhattacharya S. Ritter J. R. (1983). Innovation and communication: signalling with partial disclosure. Rev. Econ. Stud. 50, 331–346. doi: 10.2307/2297419

[ref12] Callen J. L. Fang X. (2013). Institutional investor stability and crash risk: monitoring versus short-termism? J. Bank. Financ. 37, 3047–3063. doi: 10.1016/j.jbankfin.2013.02.018

[ref300] Chemmanur T. J. He S. Hu G. (2009). The role of institutional investors in seasoned equity offerings. J. Financ. Econ. 94, 384–411.

[ref13] Chen W. Hao X. Shi N. (2014). The impact of foreign strategic investors on Bank top executives’ compensation: evidence from Chinese commercial banks. J. Financ. Res. 12, 117–132.

[ref14] Chen D. Jin Y. Dong Z. (2016). Policy uncertainty, political connection, and firms’ innovation efficiency. Nankai Bus. Rev. 19, 27–35.

[ref15] Chen L. Luo L. (2014). Research on the effect of entry barriers to foreign Investment in China—Comments on the effect of establishing China (Shanghai) pilot free trade zone. Econ. Res. J. 49, 104–115.

[ref16] Chen Q. Ma L. Yi Z. (2017). Analyst coverage and Corporate’s innovation performance: the logic of China. Nankai Bus. Rev. 20, 15–27.

[ref310] Chen M. Wang K. L. Sung Y. C. Lin F. L. Yang W. C. (2007). The dynamic Relationship between the investment behavior and the Morgan Stanley Taiwan Index: foreign institutional investors’ decision process. Rev. Pac. Basin Financ. Mark. Policies. 10, 389–413. PMID: 16594799

[ref17] Chi J. Liao J. Yang J. (2019). Institutional stock ownership and firm innovation: evidence from China. J. Multinatl. Financ. Manag. 50, 44–57. doi: 10.1016/j.mulfin.2019.04.003

[ref18] Chu D. Yang S. Song G. (2016). Fiscal subsidies, tax incentives and innovative Investment in Strategic Emerging Industry. Fin. Trade Res. 27, 83–89. doi: 10.19337/j.cnki.34-1093/f.2016.05.010

[ref19] Dai Y. Li X. Ran Z. (2019). Unequal subsidies for R&D and Enterprise innovation efficiency. Fin. Trade Res. 30, 63–78. doi: 10.19337/j.cnki.34-1093/f.2019.07.007

[ref20] Deng C. Sun J. (2014). QFII shareholding, property rights, and corporate financing constraints. J. Manag. World 05, 180–181. doi: 10.19744/j.cnki.11-1235/f.2014.05.018

[ref21] Ederer F. Manso G. (2013). Is pay for performance detrimental to innovation? Manag. Sci. 59, 1496–1513. doi: 10.1287/mnsc.1120.1683

[ref320] Elyasiani E. Jia J. J. Mao C. X. (2010). Institutional ownership stability and the cost of debt. J. Financial Mark. 13, 475–500.

[ref22] Feng G. Wen J. (2008). An empirical study on relationship between corporate governance and technical innovation of Chinese listed companies. China Industrial Econ., 91–101. doi: 10.19581/j.cnki.ciejournal.2008.07.009

[ref23] Feng Z. Wen J. (2013). Empirical research on heterogeneous institutional investor and related party transaction of firm group. Fin. Trade Res. 24, 129–137. doi: 10.19337/j.cnki.34-1093/f.2013.02.017

[ref24] Ferreira M. A. Matos P. (2008). The colors of investors’ money: the role of institutional investors around the world. J. Financ. Econ. 88, 499–533. doi: 10.1016/j.jfineco.2007.07.003

[ref330] Fu G. (2008). QFII Fund Manager Behavior Mode Surve, Huaxia Times.

[ref340] Gavious I. Hirsh N. Kaufman D. (2015). Innovation in pyramidal ownership structures. Finance Res. Lett. 13, 188–195.

[ref25] Gillan S. L. Starks L. T. (2003). “Institutional investors, corporate ownership and corporate governance: global perspectives” in Ownership and Governance of Enterprises (London: Palgrave Macmillan), 36–68. doi: 10.1057/9781403943903_2

[ref26] Graham J. R. Harvey C. R. Rajgopal S. (2005). The economic implications of corporate financial reporting. J. Account. Econ. 40, 3–73. doi: 10.1016/j.jacceco.2005.01.002

[ref27] Gu Y. Shen K. (2012). The effect of local governments’ behavior on corporate R & D investment— empirical analysis based on China’ s provincial panel data. China Industrial Econ., 77–88. doi: 10.19581/j.cnki.ciejournal.2012.10.007

[ref28] Guo Y. (2018). Signal transmission mechanism of government innovation subsidy and Enterprise innovation. China Industrial Econ., 98–116. doi: 10.19581/j.cnki.ciejournal.2018.09.016

[ref29] Hao P. Zhang X. He Y. (2020). Senior Executive's reform and opening experience and innovation decision: based on the dual adjustment effect of risk-taking and career path. South China J. Econ. 36, 108–120. doi: 10.19592/j.cnki.scje.370317

[ref31] He W. Li D. Shen J. Zhang B. (2013). Large foreign ownership and stock price informativene around the world. J. Int. Money Financ. 36, 211–230. doi: 10.1016/j.jimonfin.2013.04.002

[ref32] Holmstrom B. (1989). Agency costs and innovation. J. Econ. Behav. Organ. 12, 305–327. doi: 10.1016/0167-2681(89)90025-5

[ref33] Hou Q. Jin Q. Su L. Yu X. (2017). Lifting of short sale constraints and tunneling of controlling shareholders. China Econ. Q. 16, 1143–1172. doi: 10.13821/j.cnki.ceq.2017.02.14

[ref34] Huang W. Zhu T. (2015). Foreign institutional investors and corporate governance in emerging markets: evidence of a split-share structure reform in China. J. Corp. Finan. 32, 312–326. doi: 10.1016/j.jcorpfin.2014.10.013

[ref35] Jiang F. Shen Y. Cai X. Jiang L. (2020). Corporate governance effects of multiple blockholders: a perspective from controlling shareholders’ share pledge. J. World Econ. 43, 74–98.

[ref36] Johnson S. La Porta R. Lopez-de-Silanes F. Shleifer A. (2000). Tunneling. Am. Econ. Rev. 90, 22–27. doi: 10.1257/aer.90.2.22

[ref37] Kong D. Xu M. Kong G. (2017). Pay gap and firm innovation in China. Econ. Res. J. 52, 144–157.

[ref38] Kong D. Zhu L. Yang Z. (2020). Effects of foreign investors on energy firms' innovation: evidence from a natural experiment in China. Energy Econ. 92:105011. doi: 10.1016/j.eneco.2020.105011

[ref39] La Porta R. Lopez-de-Silanes F. Shleifer A. Vishny R. (2000). Investor protection and corporate governance. J. Financ. Econ. 58, 3–27. doi: 10.1016/S0304-405X(00)00065-9

[ref40] Lel U. (2019). The role of foreign institutional investors in restraining earnings management activities across countries. J. Int. Bus. Stud. 50, 895–922. doi: 10.1057/s41267-018-0195-z

[ref41] Li L. Han L. (2014). Value investing or value creating? A comparative study between foreign and domestic institutional investors. China Econ. Q. 13, 351–372. doi: 10.13821/j.cnki.ceq.2014.01.014

[ref42] Li C. Li Y. Li M. (2018a). Control Shareholder’s share pledge and firms ‘innovation investment. J. Financ. Res., 143–157.

[ref43] Li C. Liu B. Zhou P. Zhang X. (2018b). QFII and corporate information disclosure. J. Financ. Res., 138–156.

[ref44] Li Z. Wang B. Wu T. Zhou D. (2021). The influence of qualified foreign institutional investors on internal control quality: evidence from China. Int. Rev. Financ. Anal. 78:101916. doi: 10.1016/j.irfa.2021.101916

[ref45] Li W. Yu M. (2015). Ownership structure and corporate innovation of privatized enterprises. J. Manag. World, 112–125. doi: 10.19744/j.cnki.11-1235/f.2015.04.011

[ref46] Liu N. Bredin D. Cao H. (2020). The investment behavior of qualified foreign institutional investors in China. J. Multinatl. Financ. Manag. 54:100614. doi: 10.1016/j.mulfin.2020.100614

[ref47] Liu B. Li C. (2022). Qualified foreign institutional investor and corporate earnings management. J. Manag. Sci. 35, 97–110.

[ref48] Liu B. Wang L. (2018). Performance-based equity incentives, exercise rights vesting restriction, and corporate innovation. Nankai Bus. Rev. 17–27+38.

[ref49] Liu B. Zhao L. (2021). Does QFII improve the effectiveness of China’s capital market? J. Zhongnan Univ. Econ. Law, 79–93+160. doi: 10.19639/j.cnki.issn1003-5230.2021.0018

[ref50] Lu D. Yu D. Huang D. Yang R. (2020). Internal training and external hiring: who can better promote corporate innovation. China Industrial Econ., 157–174. doi: 10.19581/j.cnki.ciejournal.2020.10.007

[ref51] Manso G. (2011). Motivating innovation. J. Fin. 66, 1823–1860. doi: 10.1111/j.1540-6261.2011.01688.x

[ref350] Myers S. C. Majluf N. S. (1984). Corporate financing and investment decisions when firms have information that investors do not have. J. Financ. Econ. 13, 187–221.

[ref52] Peng Z. Chen X. Xu H. (2020). Research on the influence of external salary gap on Enterprise innovation efficiency. Secur. Mark. Her., 20–28.

[ref53] Romer P. M. (1990). Endogenous technological change. J. Polit. Econ. 98, S71–S102. doi: 10.1086/261725

[ref54] Shaikh I. Randhawa K. (2022). Managing the risks and motivations of technology managers in open innovation: bringing stakeholder-centric corporate governance into focus. Technovation 114:102437. doi: 10.1016/j.technovation.2021.102437

[ref55] Tan M. N. (2009). Has the QFII scheme strengthened corporate governance in China? China Int. J. 07, 353–369. doi: 10.1142/S0219747209000399

[ref56] Tang Y. Song Y. (2010). Value-selection vs. value-creation: evidence from institutional Investors in the Chinese Market. China Econ. Q. 9, 609–632. doi: 10.13821/j.cnki.ceq.2010.02.010

[ref57] Wang T. Cheng D. (2022). Executive shareholding, institutional investor shareholding and enterprise innovation. Eur. J. Innovations Manag. (Epub ahead of pre print), doi: 10.1108/EJIM-11-2021-0553

[ref58] Wang L. Kong D. Dai Y. (2018). Politicians’ promotion pressure and firm innovation. J. Manag. Sci. China 21, 111–126.

[ref59] Wang Y. Zhang G. (2018). Board networks and firm innovation: attracting resources and introducing intelligence. J. Financ. Res. 06, 189–206.

[ref60] Wen J. Feng G. (2012). Heterogeneous institutional investor, nature of firm and independent innovation. Econ. Res. J. 47, 53–64.

[ref63] Yu M. Fan R. Zhong H. (2016). Chinese industrial policy and corporate technological innovation. China Industrial Econ., 5–22. doi: 10.19581/j.cnki.ciejournal.2016.12.002

[ref64] Yu Y. Zhao Q. Ju X. (2018). Inventor executives and innovation. China Industrial Econ. 03, 136–154. doi: 10.19581/j.cnki.ciejournal.2018.03.008

[ref65] Zhang H. Ni X. (2017). How can QFII ownership affect firm innovation: evidence from patenting and R & D of listed-firms. Q. J. Financ. 11, 1–29.

